# Interleukin-35-Producing CD8α^+^ Dendritic Cells Acquire a Tolerogenic State and Regulate T Cell Function

**DOI:** 10.3389/fimmu.2017.00098

**Published:** 2017-02-08

**Authors:** Sergio Haller, Anaïs Duval, Romain Migliorini, Mathias Stevanin, Vanessa Mack, Hans Acha-Orbea

**Affiliations:** ^1^Department of Biochemistry, Center for Immunity and Infection Lausanne (CIIL), University of Lausanne, Lausanne, Switzerland; ^2^Institut de Pharmacologie et de Biologie Structurale, Centre National de la Recherche Scientifique (CNRS), Université Paul Sabatier, Université de Toulouse, Toulouse, France

**Keywords:** dendritic cells, interleukin-35, immune tolerance, EAE, mouse

## Abstract

Dendritic cells (DCs) play a central role in shaping immunogenic as well as tolerogenic adaptive immune responses and thereby dictate the outcome of adaptive immunity. Here, we report the generation of a CD8α^+^ DC line constitutively secreting the tolerogenic cytokine interleukin (IL)-35. IL-35 secretion led to impaired CD4^+^ and CD8^+^ T lymphocyte proliferation and interfered with their function *in vitro* and also *in vivo*. IL-35 was furthermore found to induce a tolerogenic phenotype on CD8α^+^ DCs, characterized by the upregulation of CD11b, downregulation of MHC class II, a reduced costimulatory potential as well as production of the immunomodulatory molecule IL-10. Vaccination of mice with IL-35-expressing DCs promoted tumor growth and reduced the severity of autoimmune encephalitis not only in a preventive but also after induction of encephalitogenic T cells. The reduction in experimental autoimmune encephalitis severity was significantly more pronounced when antigen-pulsed IL-35^+^ DCs were used. These findings suggest a new, indirect effector mechanism by which IL-35-responding antigen-presenting cells contribute to immune tolerance. Furthermore, IL-35-transfected DCs may be a promising approach for immunotherapy in the context of autoimmune diseases.

## Introduction

The immune system has to cope simultaneously with foreign substances while maintaining tolerance to self-antigens. Dendritic cells (DCs) play a dual role in linking and integrating innate and adaptive immune responses. Due to their central role, DCs are particularly interesting targets for novel immunotherapeutic approaches for the specific treatment of autoimmune diseases.

Interleukin (IL)-35 is a heterodimeric protein composed of a P35 (*Il12a*) and an Ebi3 (*Il27b*) chain. IL-35 is described to be produced predominantly by natural regulatory T (Treg) as well as CD138^+^ plasma cells (1, 2). Administration or over-expression of IL-35 has been shown to confer protection to the development of various experimental autoimmune diseases through the suppression of CD4^+^ T cell proliferation and function. IL-35 furthermore induces a distinct subset of Foxp3^−^ Treg (iTr35) and B cells (1, 2). More recently, IL-35 has been shown to be involved in the maintenance of tolerance in the intestinal tract (1, 3). In addition, EBI3, but not IL-27p28, is expressed in different human cancers like Hodgkin lymphoma or nasopharyngeal carcinoma cells (4, 5). IL-35 production has not only been implicated in neoplastic but also in tumor-associated cells, suggesting a more diverse production and function than initially proposed (6, 7). Despite these recent insights, the biological relevance for immunoregulation as well as the exact function of the cytokine IL-35, especially on non-lymphoid cells, remains elusive.

We previously described a stable CD8α^+^ DC line [murine tumor DC line (MuTu DC)] (8, 9). This cell line exhibits phenotypical and functional characteristics of freshly isolated CD8α^+^ DCs, i.e., expression pattern of surface markers, potent costimulation, cross-presentation of antigens, and induction of cytotoxic responses by CD8^+^ T lymphocytes (9). MuTu DCs can easily be cultured for over a time span of around 40 passages *in vitro*. The MuTu DCs exhibit an immature phenotype with low expression of costimulatory molecules, which can be upregulated upon activation with toll-like receptor ligands. Intravenously injected MuTu DCs accumulate primarily in liver and spleen. They are, however, rejected in a CD8^+^ T cell-dependent manner within 5 days after transfer. By contrast, adoptive transfer into *rag*-deficient or SV40lgT transgene tolerant mice as well as into wild-type mice depleted of CD8 T cells leads to development of MuTu DC tumors within 1 month (10). The MuTu cell line allowed us to investigate the tolerogenic potential of DCs in a rational and standardized way. Our results demonstrate that the expression of IL-35 potently regulates CD4^+^ as well as CD8^+^ T cell responses *in vitro* and also in *in vivo*. We could further show that CD8α^+^ DCs respond to IL-35 signaling by acquiring a tolerogenic phenotype. Antigen pulsing of the IL-35-expressing MuTu DC line was further shown to ameliorate autoimmune encephalitis in a preventive as well as in a therapeutic setting. Our experiments indicate this effect to be, at least partially, dependent on interactions with IL-35-conditioned DCs. These findings suggest a novel, indirect mechanism by which IL-35 modulates adaptive immune responses.

## Animals and Methods

### Mice

C57BL/6, BALB/c, and RAG1^−/−^ C57BL/6 mice were purchased from Harlan laboratories. OT-I and OT-II transgenic mice were maintained and bred in our own facility. All animals were kept and bred in a specific pathogen free animal facility. For all experiments, female, age-matched (6–8 weeks) mice were used. All animal experiments were performed after approval by the cantonal veterinary office (Service de la consommation et des affaires vétérinaires, Département du territoire et de l’environnement, Permission no. VD2490.1).

### Generation of IL-35^+^ MuTu DC Line

Murine tumor DC lines are derived from splenic tumors isolated from transgenic CD11c:SV40LgT C57BL/6 mice (8). Their phenotypical and functional characteristics have been thoroughly described by Fuertes Marraco et al. (9) and Duval et al. (10). A single-chain IL-35 construct, consisting of *Ebi3* (*Il27b*) and *p35* (*Il12a*), linked by (Gly_4_Ser)_3_ was amplified from mouse DCs, respectively, IL-12 expression vector (courtesy by B. Becher, Zürich) and cloned into pRRLSIN.cPPT.PGK-GFP.WPRE lentiviral expression vector (courtesy by D. Trono, Lausanne). The reading frame of *Ebi3*-(Gly_4_Ser)_3_-*P35* was sequenced to exclude mutations. Lentiviral particles containing either an empty or an IL-35 expression vector were produced in 293T HEK cells. Wild-type MuTu DC line was stably transduced with either empty vector (mock DC) or IL-35 lentiviral particles (IL-35^+^ DC) (MOI = 20) and the cells maintained *in vitro* for maximally 15 passages. Transgene expression was confirmed after each transduction by RT-PCR using the following primers: *Ebi3*: 5′-ATGTCCAAGCTGCTCTTCCT-3′ (forward), 5′-AGAGGAGTCCAGGAGCAGTC-3′(reverse). *P35*: 5′-AAATGAAGCTCTGCATCCTGC-3′(forward), 5′-TCACCCTGTTGATGGTCACG-3′ (reverse). All MuTu DC lines were cultured in IMDM–Glutamax (GIBCO) supplemented with 10% heat-inactivated fetal calf serum, 10 mM HEPES (GIBCO), 50 µM β-mercaptoethanol (GIBCO), 50 U/ml of penicillin, and 10 µg/ml streptomycin (GIBCO) at 37°C in 5% CO_2_ atmosphere.

### IL-35 Immunoprecipitation

Interleukin-35 was precipitated from cell lysate using IL-12p35-specific antibodies (R&D systems, clone #45806) and protein A/G beads (Santa Cruz Biotechnology). Western blotting was conducted as described previously. In brief, cell lysates were centrifuged at 12,000 rpm for 30 min at 4°C. Protein concentrations of cell lysates were determined with the BCA assay (Pierce) and 40 µg of proteins were loaded onto 7.5–15% SDS-polyacrylamide gels. The gels were transferred to nitrocellulose membrane (Amersham Pharmacia Biotech). The blot was blocked in blocking buffer (5% non-fat dry milk/1% Tween 20 in PBS) for 2 h at room temperature and then incubated with Ebi3-specific antibody (Shenandoah Biotechnology, clone V1.4H6.29) in TBST overnight at 4°C. The blot was incubated with HRP-coupled secondary antibody for 2 h at room temperature. Blot was visualized by ECL (Pierce Biotechnology).

### T Cell Proliferation Assays

#### Mixed Leukocyte Reaction (MLR)

BALB/c responder cells were isolated from spleen and popliteal lymph nodes using 40-µm cell strainer. CD4^+^ and CD8^+^ T cells were purified by immunomagnetic bead-based sorting following protocol supplied by the manufacturer (Miltenyi Biotec). Then, 1 × 10^5^ responder T cells were stained with 5 µM efluor670 proliferation dye (eBioscience) and cocultivated with either control DCs, IL-35^+^ DCs, or syngeneic CD4^+^ or CD8^+^ depleted splenocytes at different ratios in 96-well U-bottom plates. T cell proliferation and activation were assessed after 3 days by flow cytometry and IL-35 mRNA expression was measured by RT-PCR.

#### iTreg Suppression Assay

Suppression assays were performed as described by Collison and Vignali (11). Briefly, 1 × 10^5^ IL-35^+^ DCs or mock DCs were plated in a 24-well plate and incubated overnight. DCs were activated with CpG and polyI:C for 6 h. CD4^+^ T cells were magnetically isolated from C57BL/6 mice using the naïve CD4^+^ T cell isolation kit (Miltenyi) and plated at a 10:1 (1 × 10^6^ cells) ratio to mock or IL-35^+^ DCs. A 2 ng/ml recombinant TGF-β and a 10 ng/ml IL-2 were added to some of the wells containing mock DCs for the generation of Foxp3^+^ Tregs. Cells were incubated for 4 days. Different amounts of the generated suppressor T cells were then seeded with 2.5 × 10^4^ efluor670 proliferation dye stained naïve target CD4^+^ T cells. Proliferation of the target cells was assayed after 3 days. Percent suppression was calculated using the following formula:

$$\frac{\%\text{ proliferated Tconv alone}-\%\text{ proliferated Tconv treated with Treg}}{\text{\% proliferated Tconv alone}}\times 100.$$

#### *In Vitro* OT-I/II Proliferation

The 5 × 10^4^ cells of the respective MuTu DC line were pulsed for 4 h with different concentrations of ovalbumin-specific peptides SIINFEKL (OT-I) or OVA_329–337_ (OT-II). CD8^+^ or CD4^+^ T cells were magnetically isolated from OT-I/Rag^−/−^ or OT-II/Rag^−/−^ mice, respectively and stained with 5 µM efluor670 proliferation dye (eBioscience). Then, 1 × 10^5^ T cells were cocultured with the DCs for 3 days when the non-adherent T cells were harvested and analyzed by flow cytometry and real-time PCR.

#### *In Vivo* OT-I/II Proliferation

CD8^+^ or CD4^+^ T cells were magnetically isolated from CD45.1^+^ OT-I/Rag^−/−^ or OT-II mice, respectively and stained with 5 µM efluor670 proliferation dye (eBioscience). The 2 × 10^5^ T cells were intravenously injected into CD45.2 C57BL/6 mice. Then, 2.5 × 10^6^ cells of the respective CD8α^+^ DC line were pulsed with either SIINFEKL (OT-I restricted, 1 µg/ml) or OVA_329–337_ (OT-II restricted, 50 µM) peptide and were i.v. injected on the same day and 1 day after T cell transfer. Mice were sacrificed 4 days later and the isolated splenocytes were analyzed by flow cytometry.

### Tumor Experiments

B16.F0 melanoma and CMT93 colon carcinoma tumor cell lines were cultured in DMEM medium (GIBCO), supplemented with 10% FCS, and 50 U/ml of penicillin and 50 mg/ml streptomycin (GIBCO). Tumor cell lysate was generated by five consecutive freeze/thaw cycles in liquid nitrogen. Lysate was centrifuged at 1500 g for 15 min before use. Mock- or IL-35-transduced DCs were pulsed with respective lysate in a 1:4 lysate to DC ratio and 100 U/ml IFN-γ (eBioscience). Five and three days before and three days after tumor cell transfer, 2.5 × 10^6^ DCs were s.c. injected in the flank. Tumor was induced by s.c. injection of 2 × 10^5^ B16.F0 or 2 × 10^6^ CMT93 cells into the same flank. Tumor growth was followed by measuring length and width using a caliper. Tumor volume was measured using the formula $V=\frac{\pi }{6}\cdot 1.58\cdot {\left(length\cdot width\right)}^{\frac{3}{2}}$ as described by Wurzenberger et al. (12).

### Flow Cytometry

The fluorochrome-conjugated antibodies used were specific to CD11c (clone N418, PECy7), GR1 (clone RB6-8C5, PE, or PerCP-PECy5.5), CD8a (clone 54-6.7, FITC, or APC-Cy7), DEC205 (clone 205yekta, PerCP-eF710), CD24 (cloneM1/69, PerCP-Cy5.5), Clec9A (clone 42D2, PE), CD11b (clone M1/70, APC), CD3 (clone 500A2, ef450), CD4 (clone RM4-5, APC, eF450), MHCI (clone AF6-88.5.5.3, APC), MHCII (clone M5/114.15.2, PE), CD40 (clone 1C10, APC), CD80 (clone 16-10A1, PECy5), CD86 (clone GL1, APC, or Alexa750), panNK (clone DX5, FITC), Ly6C (clone HK1.4, APC), and Ly6G (clone 1A8, PerCP-Cy5.5). For intracellular staining, the T cells were restimulated for 4 h with 10 ng/ml PMA and 500 ng/ml Ionomycin, in the presence of 10 µg/ml Brefeldin A. After extracellular staining, cells were fixed for 30 min in 4% paraformaldehyde and permeabilized in 0.5% Saponin buffer for 30 min. Intracellular staining was performed in Saponin buffer for 30 min. All antibodies were acquired from eBioscience or Biolegend. Flow cytometric analyses were performed on LSRII cytometer (Becton Dickinson) using FACS Diva6 (Becton Dickinson) and FlowJoX (Tree Star) software for data processing.

### Induction of Adoptive Transfer Experimental Autoimmune Encephalitis (EAE)

Adoptive transfer EAE was induced as described by Miller and Karpus (13). Briefly, myelin oligodendrocyte glycoprotein (MOG) reactive T cells were primed in donor C57BL/6 mice by two sub-cutaneous injections of MOG_35–55_-peptide emulsified in complete Freud’s Adjuvant (Sigma). Intraperitoneal injection of 200 ng Pertussis Toxin (Sigma) was used to boost lymphocyte priming. In order to study the effect of tolerogenic molecules, MOG_35–55_ peptide-pulsed DCs were intravenously injected 1 day before and 1 day after the immunization. Twelve days post immunization, inguinal, brachial, and axillary lymph nodes were isolated by digestion in 1 mg/ml Collagenase D (Roche) for 20 min at 37°C. Single cells suspension was prepared using a 40-µm cell strainer (Falcon). Lymph node cells were expanded *in vitro* for 3 days in the presence of MOG_35–55_-pulsed DCs and 0.5 ng/ml recombinant IL-12p70 (eBioscience). Then, 5 × 10^6^ MOG-specific T cells were then transferred to C57BL/6 recipient mice by i.p. injection. Mice were i.v. injected with MOG_35–55_-pulsed DCs 1 day after T cell transfer. Clinical EAE symptoms were scored daily in a blinded manner: 0—no obvious changes in motor functions; 1—decreased tail tonus; 2—abnormal gait (ataxia and/or impaired righting reflex); 3—partial hind limb paralysis; 4—complete hind limb paralysis; and 5—complete hind and fore limb paralysis.

### Gene Expression Analysis by Real-time PCR

Total RNA was isolated from the cells and purified using the RNeasy^®^ spin columns following the manufacturer’s protocol (Qiagen). Total RNA yields were quantified by Nanodrop spectrophotometry (Thermo Fisher Scientific). Synthesis of cDNA was performed using random nonamer primers and the Superscript II Reverse Transcriptase kit (Invitrogen). cDNA was purified using the PCR clean-up kit (Qiagen) and stored at −80°C.

Real-time PCR (RT-PCR) was performed in technical triplicates using SYBR FAST qPCR mix (KapaBiosystems) and 5 ng RNA/reaction on a LightCycler480 machine (Roche Diagnostics). Relative expression levels were analyzed using qBase+ 1.2 data analysis software (BioGazelle). Gene expression for each individual sample was normalized to the housekeeping genes HPRT 5′-AGCCTAAGATGAGCGCC-3′ (forward) and 5′-TTACTAGGCAGATGGCCACA-3′ (reverse) and β-actin 5′-GCACAGCTTCTTTGCAGCTCCTTCG-3′ (forward) and 5′-TTTGCACATGCCGGAGCCGTTG-3′ (reverse).

### Statistical Analysis

Differences between groups were determined using either unpaired two-tailed Student’s *t*-test or Mann–Whitney *U* test where indicated. All statistical analyses were performed using Prism 6.0 (GraphPad). *p*-Values are indicated when considered statistically significant (*p ≤ 0.05, **p ≤ 0.005, ***p ≤ 0.001).

## Results

### IL-35 Inhibits Upregulation of MHC Class II and Costimulatory Molecules by CD8α^+^ DCs upon Activation

A stable murine DC line, constitutively expressing a biologically functional, single-chain IL-35 construct (Supplementary Figure 1A) was generated by lentiviral transduction of the recently established CD8α^+^ MuTu DC line (thereafter named “wtDC”) (9). *Il12p35* and *Ebi3* transcripts were detected *via* quantitative PCR (Supplementary Figure 1B). Ebi3 protein expression was confirmed by western blot of the cell lysate of cultured IL-35-expressing DCs after immunoprecipitation using anti-IL-12p35 antibody (Supplementary Figure 1C). For the remainder of this manuscript, the IL-35-expressing MuTu DC line is referred to as “IL-35^+^ DC.”

**Figure 1:**
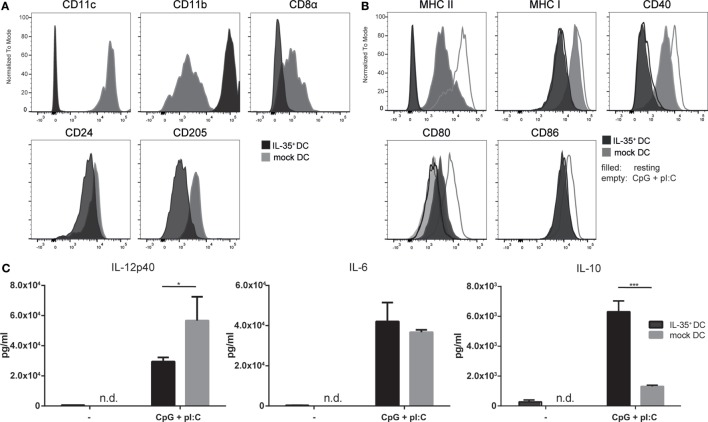
**Interleukin (IL)-35-transduced dendritic cells (DCs) upregulate CD11b and do not respond to activation stimuli**. IL-35- and mock-transduced DCs were cultured under equal conditions and analyzed by flow cytometry for the expression of panel **(A)** DC-specific markers or panel **(B)** activation markers upon 12 h stimulation with 5 µg/ml CpG and 1 µM pI:C. Data representative of four independent experiments. **(C)** IL-35- or control-transduced DCs were seeded and incubated for 3 days. The cells were stimulated as described above for the last 12 h or not and the secretion of the indicated cytokines was quantified by ELISA. Results indicated as the mean of biological triplicates ± SD. Data representative for one of six independent experiments.

Additionally, empty vector-transduced DCs (mock DCs) were generated and used as controls when appropriate. We could not observe any noticeable differences in the morphological characteristics or the proliferative capacity when comparing the wtDC line to the lentivirally transduced MuTu DC lines. Six independent transductions of MuTu DCs were conducted yielding IL-35-expressing DCs with comparable functional and phenotypic properties. The IL-35^+^ MuTu DC cell line was assessed for the expression of a panel of surface markers commonly used to characterize CD8α^+^ DCs by direct comparison to mock DCs. Flow cytometric analysis revealed a marked reduction of CD11c, CD8α, and DEC205 surface expression on IL-35^+^ DCs. By contrast, the expression of CD11b was drastically increased (Figure 1A). Furthermore, the IL-35-expressing DC line exhibited lower expression of MHC I and MHC II as well as costimulatory molecules CD40 and CD86 (Figure 1B). Activation of the control DCs with TLR3 and TLR9 ligands polyI:C and CpG led to increased surface expression of MHC II as well as costimulatory receptors CD40, CD80, and CD86 by control DCs. Conversely, IL-35^+^ DCs exhibited a complete unresponsiveness to TLR stimulation as indicated by the failure to upregulate any of the analyzed (co-)stimulatory molecules. Interestingly, IL-35^+^ DCs showed an increased CD80 expression already under resting condition when compared to mock DCs. A more than twofold reduction in IL-12p40 secretion could be detected by ELISA in the culture medium of IL-35^+^ DCs while the production of the immunoregulatory cytokine IL-10 was found to be increased significantly (Figure 1C).

**Figure 2:**
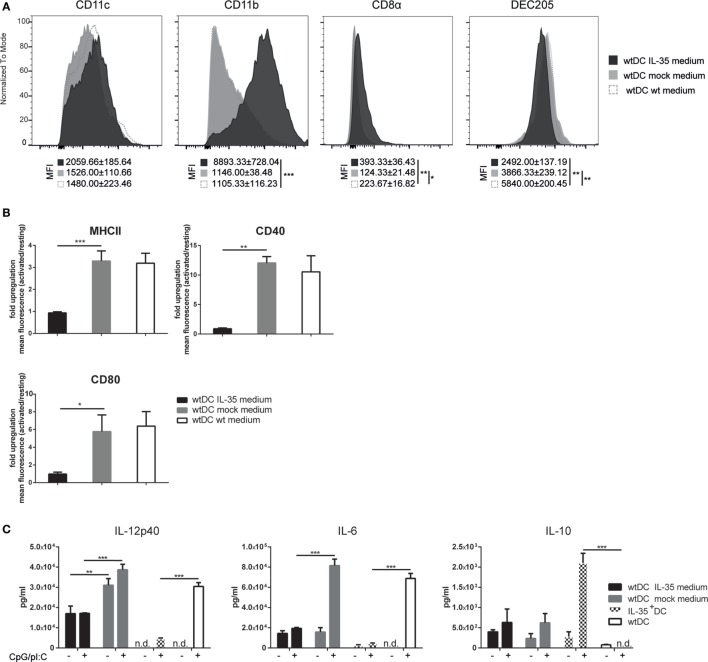
**Interleukin (IL)-35 abrogates the immunogenicity of murine tumor DC lines (MuTu DCs)**. Wild-type MuTu DC line was cultured for 4 days in the presence of filtered and concentrated supernatant of IL-35 vector-transfected 293T HEK cells. wtDCs incubated with either empty vector-transfected 293T HEK cells or normal medium served as controls. **(A)** Dendritic cells (DCs) were analyzed for expression of DC-specific markers by flow cytometry. **(B)** wtDCs were cultured as described above and activated with 5 µg/ml CpG and 1 µM pI:C (light gray histograms) or not (black histograms). Bar charts summarize fold upregulation of the respective activation marker upon activation. Experiments were conducted in biological triplicates and results are shown as the mean of the ratio of geometric mean activated cells/geometric mean resting cells ± SD. MFI, mean fluorescence intensity. Data representative for one of five independent experiments. **(C)** Wild-type MuTu DCs were conditioned and activated as described above. The cytokine secretion in the culture medium was assayed by ELISA. Data representative for one of three independent experiments. n.d., not detectable. Results depicted as the mean of biological triplicates ± SD.

In order to investigate whether the observed phenotypic change of IL-35^+^ DCs is provoked by an autocrine effect of the transgene, wild-type MuTu DCs were cultured for 4 days in IL-35 containing culture medium. As observed for IL-35-transduced MuTu DCs, IL-35-conditioned wild-type DCs expressed markedly higher levels of CD11b. On the contrary, the expression of CD11c, CD8α, and DEC205 was not significantly altered when compared to cells cultured in control medium (Figure 2A). Upon activation with TLR3 and TLR9 ligands, IL-35-conditioned but not control DCs exhibited a significantly impaired upregulation of MHCII and the costimulatory receptor CD40. Similar to IL-35^+^ DCs, IL-35 conditioning increased the expression of CD80 already under steady-state conditions and TLR stimulation did not further augment its surface expression (Figure 2B). Moreover, upon TLR stimulation, MuTu DCs cultured in IL-35 supplemented medium secreted significantly less IL-12p40 and IL-6 cytokine when compared to DC grown in control medium. In contrast to IL-35^+^ DCs, no increase in IL-10 secretion could be detected (Figure 2C).

### IL-35-Expressing MuTu DCs Impair T Cell Proliferation and Function

**Figure 3:**
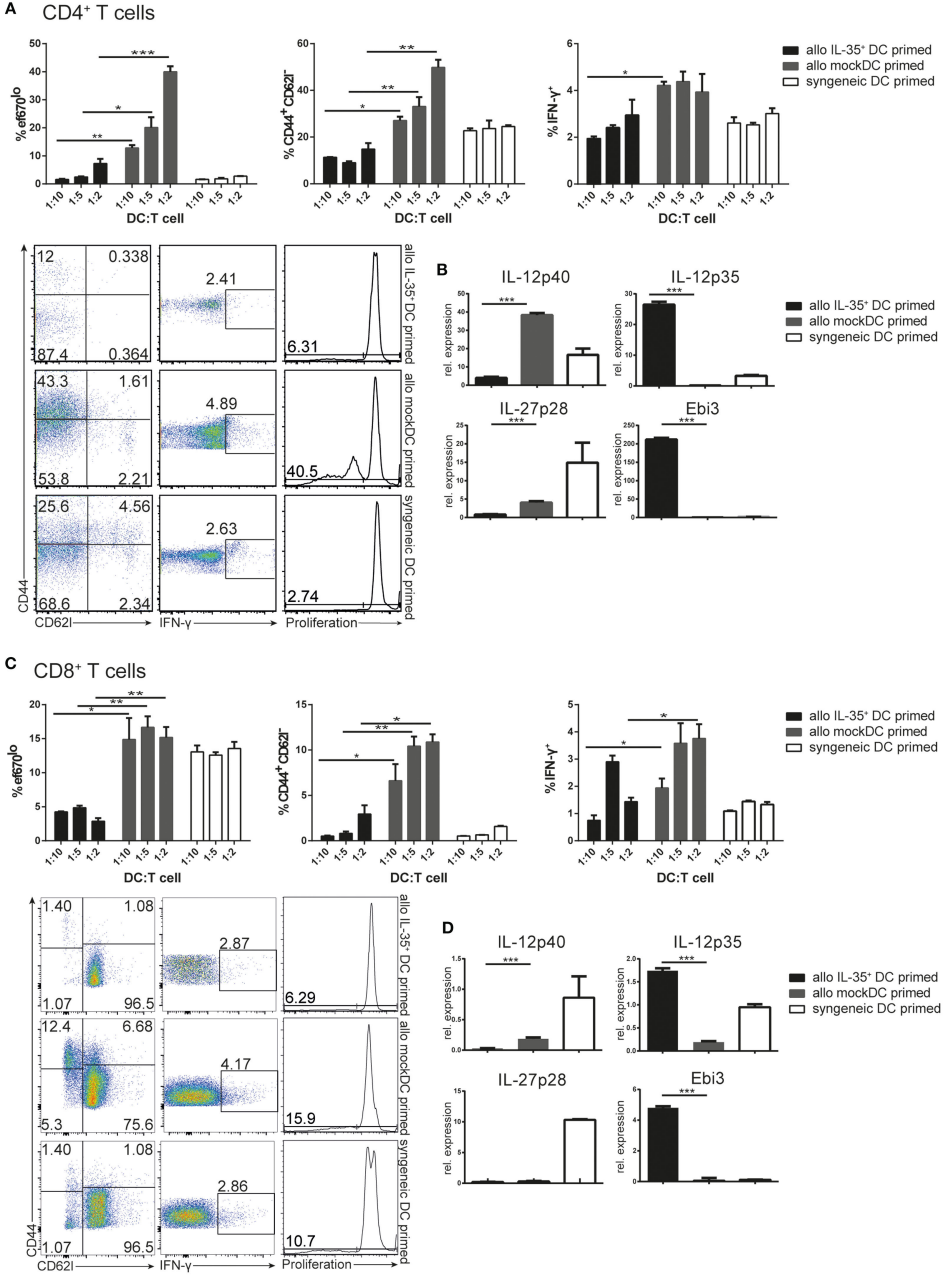
**Allogeneic stimulation of BALB/c T cells with C57BL/6 interleukin (IL)-35^+^ dendritic cells (DCs)**. Naïve CD4^+^ T **(A,B)** or CD8^+^ T **(C,D)** cells were isolated and magnetically purified from BALB/c mice and cultured for 3 days at different ratios with C57BL/6-derived mock transduced or IL-35^+^ murine tumor DC lines (allogeneic priming) or syngeneic wild-type DCs. **(A,C)** Proliferation, IFN-γ, and CD44/CD62L expression of the T cells was analyzed by flow cytometry after 3 days. **(B,D)** T cells were isolated and the transcription of IL-12 cytokine family-associated genes was assessed by real-time PCR. Results representative of three **(A,B)** and two **(C,D)** independent experiments. Data are depicted as mean ± SD.

We have previously demonstrated that the MuTu DC lines efficiently induce T cell proliferation in MHCI or MHCII restricted systems (9). IL-35 on the other hand, is described to inhibit CD4^+^ T cell proliferation (2, 14). We therefore tested whether the IL-35-producing DC line is able to affect T cell activation and function. An allogeneic MLR was performed using variable amounts of mock transduced or IL-35 expressing, C57BL/6-derived DCs as stimulators. Coculture with mock-transduced DCs resulted in a robust, DC number-dependent proliferation of BALB/c-derived CD4^+^ responder T cells. This was accompanied by an increased percentage of activated CD44^+^ CD62L^−^ T cells and IFN-γ-expressing T cells. On the contrary, target cell proliferation and activation were severely reduced when IL-35^+^ DCs were used as activator cells (Figure 3A). Analysis of target T cell RNA revealed a significant upregulation of IL-35-associated *P35* and *Ebi3*, but not IL-12- or IL-27-specific *P40*, respectively, P*28* transcripts. In contrast to IL-35^+^ DCs, culture with mock-transduced DC led to a significant upregulation with IL-12/23-associated *P40* but not *P35* or *Ebi3* transcripts (Figure 3B). No single-chain IL-35 cDNA could be detected by PCR, excluding the possibility of a contamination of the RNA by IL-35^+^ DCs (Supplementary Figure 2). Similar results were obtained upon the cotransfer of OVA_323–339_ peptide-pulsed mock or IL-35^+^ DCs and congenically marked OT-II CD4^+^ T cells into C57BL/6 mice. Four days after the transfer, IL-35-expressing but not control-transduced DCs were shown to reduce both the amount of detectable CD45.1^+^ OT-II T cells as well as their IFN-γ production (Supplementary Figure 3A).

There is only limited information on the effects of IL-35 on the proliferation and effector functions of CD8^+^ T cells available. Allogeneic MLR-culturing BALB/c-derived CD8^+^ T cells with C57BL/6 IL-35^+^ or mock-transduced MuTu DCs were therefore performed. We found that IL-35^+^ but not mock DCs significantly reduced proliferation, activation status, as well as IFN-γ expression by CD8^+^ T lymphocytes (Figure 3C). IL-35^+^ DCs furthermore induced the transcription of significantly higher levels of *Ebi3* and *P35* in CD8^+^ T cells than mock-transduced DCs (Figure 3D). Similarly, a drastic reduction in the abundance of CD45.1^+^ CD8^+^ T cells could be observed upon co-injection of OT-I CD8^+^ T cells together with OVA_323–339_ peptide-pulsed IL-35-expressing but not mock-transduced DCs into C57BL/6 mice (Supplementary Figure 3B). No differences in cell number and in IFN-γ expression of host-derived CD45.2^+^ CD8^+^ T cells were observed (Supplementary Figure 3C).

**Figure 4:**
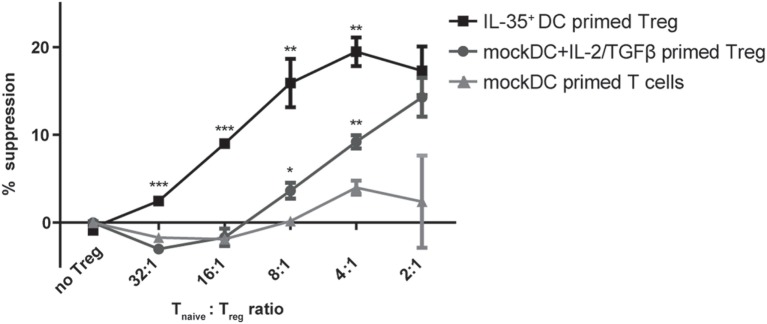
**Interleukin (IL)-35^+^ dendritic cell (DC) cocultured T cells suppress target T cell proliferation**. Naïve CD4^+^ T cells were primed in the presence of IL-35^+^ DCs or mock DCs and 10 ng/ml IL-2/2 ng/ml TGF-β for 4 days. The suppressive capacity of the primed T cells was then assayed by culturing for 3 days them with naïve CD4^+^ responder T cells in the presence of anti-CD3/CD28 stimulation. Results are indicated as mean percent of suppression ± SD. Data representative of four independent experiments.

Interleukin-35 cytokine was originally described to mediate infectious tolerance *via* the induction of a regulatory phenotype on naïve CD4^+^ T cells (14). We therefore tested whether the observed induction of *P35*/*Ebi3* transcription by CD4^+^ T cells cocultured with IL-35^+^ DCs is accompanied with the acquisition of suppressive properties. Purified naïve wild-type CD4^+^ T cells were cocultured for 4 days with either IL-35^+^ DC or mock DCs and recombinant IL-2 and TGF-β. A classical suppression assay was then performed by culturing the conditioned T cells at different ratios with naïve CD4^+^ responder T cells in the presence of CD3/CD28 stimulation. We could measure a significantly reduced, ratio-dependent, responder cell proliferation when suppressor T cells were conditioned in the presence of IL-35^+^ DC or mock DC supplemented with IL-2 and TGF-β. No such effect was observed when cells were conditioned with mock DC alone. However, IL-35^+^ DC-conditioned T cells consistently proved to be more potent suppressors than TGF-β/IL-2-induced Tregs. Both in terms of maximal suppressive capability as well as the minimal ratio of suppressor T cells needed to observe an inhibitory effect. In accordance with literature, IL-35 induced regulatory cells did not express the classical Treg cell marker Foxp3. T cells conditioned for 4 days in IL-35 containing medium but in the absence of mock DC, did not exert any suppressive functions (Figure 4 and data not shown).

### IL-35-Conditioned MuTu DCs Impair CD4^+^ T Cell Proliferation and Function

**Figure 5:**
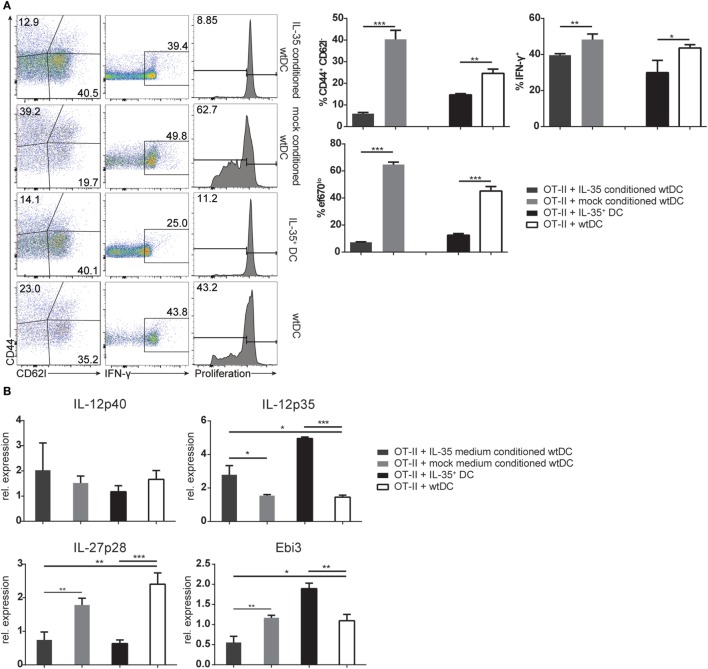
**Interleukin (IL)-35-conditioned wild-type dendritic cells (DCs) impede T cell function**. Wild-type murine tumor DC lines were conditioned in IL-35 containing or control culture medium. After 3 days, the DCs were pulsed with 50 nM OVA_323–339_ peptide and cultured with CD4^+^ OT-II T cells in the absence of IL-35. **(A)** OT-II cells were harvested after 3 days, and proliferation, IFN-γ production, and activation were assessed by flow cytometry. **(B)** The transcription of IL-12 cytokine family-associated genes by the OT-II cells was analyzed by real-time PCR. The data represent the mean ± SD of biological triplicates of one of three independent experiments.

As demonstrated above, IL-35^+^ DCs as well as wild-type DCs cultured in IL-35 containing medium did not adequately upregulate MHCII and costimulatory receptors upon activation, but remained in a rather immature state with increased CD80 expression. Such immature DC populations have been described to induce and maintain T cell tolerance (15). We therefore considered the possibility that the observed effects on T cells were not only caused directly by the secreted IL-35, but indirectly through the modulation of the immunogenic potential of the conditioned DCs. In order to test this hypothesis, wild-type MuTu DCs were conditioned for 3 days in IL-35 containing or control culture medium. Upon removal of IL-35, OVA_323–339_-pulsed IL-35-conditioned DCs exhibited a significantly impaired potential to stimulate OT-II CD4^+^ T cell proliferation, activation, and IFN-γ production when compared to control medium-conditioned MuTu DCs. The inhibitory effect on CD4^+^ T cells by IL-35-conditioned DCs was found to be comparable to OT-II CD4^+^ T cells stimulated with IL-35^+^ DCs (Figure 5A). In contrast to coculture with IL-35-expressing DCs, no induction of *Ebi3* transcription and only a moderate induction of IL-12p35 were observed by OT-II CD4^+^ T cells incubated with IL-35-conditioned wtDC. Interestingly, IL-35-conditioned DCs as well as IL-35^+^ DCs transcribed significantly less of the IL-27-associated *p28* chain than control DCs (Figure 5B).

Taken together, the inhibitory effect on T cell proliferation and function *in vitro* and *in vivo* demonstrated that IL-35 transgenic DC line efficiently interfered with proliferation of both, CD4^+^ and CD8^+^ T cells. Furthermore, IL-35^+^ DC impaired the function of CD4^+^ T cells. Our findings further indicate that IL-35 secretion alone does not only directly affect T cell proliferation and function but also indirectly *via* the induction of a tolerogenic phenotype on MuTu DCs.

### Vaccination with IL-35^+^ DCs Promotes Tumor Growth

**Figure 6:**
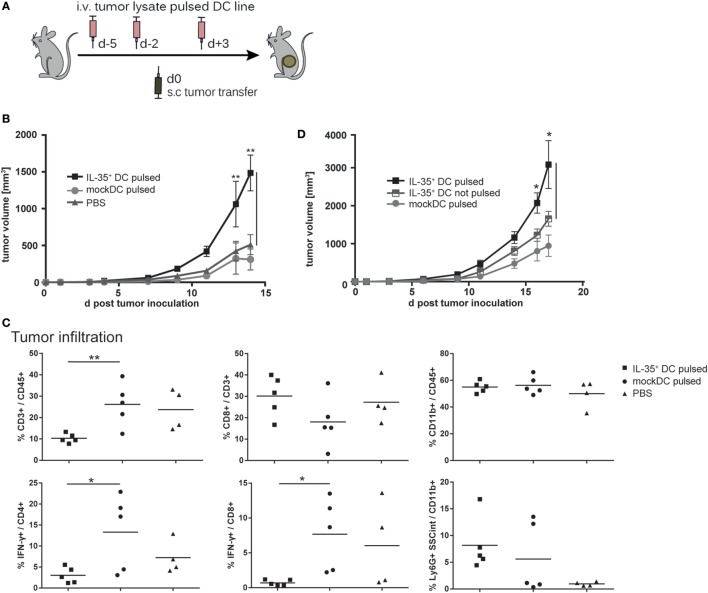
**Interleukin (IL)-35-expressing dendritic cells (DCs) promote tumor growth**. **(A)** C57BL/6 mice were intravenously vaccinated a total of three times with 2.5 × 10^6^ tumor cell lysate pulsed, IFN-γ-activated IL-35^+^ DCs, unpulsed IL-35^+^ DCs, mock DCs, or PBS. Then, 2 × 10^5^ B16F0 melanoma cells were transferred subcutaneously in the flank of the animals. **(B,D)** Tumor growth was measured regularly using a caliper. Results are expressed as the mean of tumor volumes ± SD from one of three **(B)** and two **(D)** independent experiments (*n* = 5 per experimental group). Tumor volume in mice that received tumor lysate pulsed IL-35^+^ DCs was significantly increased when compared to panel **(B)** pulsed mock DC or panel **(D)** unpulsed IL-35^+^ DC (*p ≤ 0.05, Mann–Whitney *U* test). **(C)** Tumors were resected 10 days after inoculation and enzymatically digested. The isolated cells were restimulated and tumor infiltrating cells analyzed by flow cytometry. The data show representative results from individual mice from one of three independent experiments (*n* = 5 per experimental group).

Interleukin-35 has been shown to promote tumor progression by interfering with tumor-directed immune responses in several human cancers (6, 16, 17). In order to determine whether IL-35^+^ DCs affect T lymphocyte-dependent antitumor responses *in vivo*, C57BL/6 mice (*n* = 5 per group) were vaccinated a total of three times with tumor cell lysate pulsed, IFN-γ-stimulated DCs. Five days after first vaccination 2 × 10^5^ B16.F0 melanoma cells were transferred subcutaneously into the flank of the mice (Figure 6A). B16.F0 tumor volume increased progressively and withdrawal criteria were reached in median within 20.5 days in PBS-injected mice but only 14 days for mice vaccinated with IL-35^+^ DCs. Mice that were vaccinated with control DCs exhibited a slight, but not significant delay in tumor growth when compared to PBS-injected animals (Figure 6B). Flow cytometric analysis of tumor tissue showed drastic reduction of tumor infiltration by CD3^+^ T cells in IL-35^+^ DC-vaccinated mice. In addition, infiltrating CD4^+^ and CD8^+^ T cells exhibited a significant reduction in IFN-γ production. However, no differences in Foxp3^+^ Treg cells infiltration could be observed (data not shown). IL-35^+^ DC transfer did not significantly affect the percentage or composition of infiltrating CD11b^+^ myeloid cells (Figure 6C). We next sought to determine whether IL-35-conditioned DCs contribute to the accelerated increase in tumor volume. B16.F0 growth was compared upon vaccination with either unpulsed or tumor lysate pulsed, IFN-γ-activated IL-35^+^ DCs. Only the transfer of tumor lysate loaded IL-35^+^ DCs but not unpulsed IL-35^+^ DCs caused a significant increase in tumor volume (Figure 6D).

Similarly, subcutaneously transferred CMT93 carcinoma cells showed progressive tumor growth in IL-35^+^ DC-vaccinated mice and tumor volume was significantly bigger when compared to control animals (Supplementary Figure 4A). In accordance to our observations in the B16.F0 model, flow cytometric analysis of CMT93 tumor infiltrating leukocytes revealed a markedly decreased accumulation of total CD3^+^ T lymphocytes in IL-35^+^ DC-treated mice. Both CD4^+^ and CD8^+^ T cells produced significantly less IFN-γ. In contrast to the B16.F0 model, vaccination with IL-35^+^ DCs was furthermore accompanied with an increased infiltration by CD11b^+^ Gr1^+^ myeloid-derived suppressor cells when compared to control DC-vaccinated animals (Supplementary Figure 4B).

### Transfer of IL-35^+^ MuTu DCs Interferes with Development of EAE

**Figure 7:**
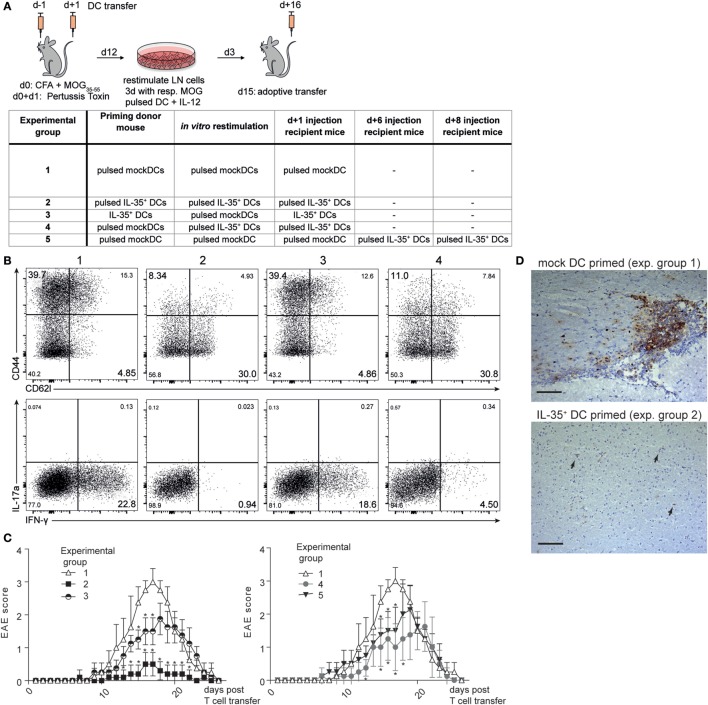
**Interleukin (IL)-35-expressing dendritic cells (DCs) ameliorate experimental autoimmune encephalitis (EAE) pathogenesis**. **(A)** Myelin oligodendrocyte glycoprotein (MOG)-specific T cells were primed in the presence of the respective DC lines in C57BL/6 donor mice. Lymph node cells were harvested after 12 days and restimulated *in vitro* for 3 days in the presence of the respective MOG-pulsed DC line and IL-12. Then, 5 × 10^6^ T cells were transferred into recipient C57BL/6 mice. And then, 2.5 × 10^6^ of the respective DCs were injected i.v. 1 day later. The table summarizes the different treatment regimens for each experimental group **(B)** Restimulated CD3^+^ CD4^+^T cells were analyzed by flow cytometry for the expression of CD44/CD62L and IFN-γ/IL-17a. Shown are representative plots of one of three independent experiments. **(C)** Recipient mice were examined daily using a clinical EAE scoring system ranging from 0 (no symptoms) to 5 (complete paralysis). For the sake of clarity, the data of one representative experiment out of four are shown in two graphs (*n* = 5 animals per experimental group). Data denoted as mean ± SD. *p ≤ 0.05 against control group 1. Mann–Whitney *U* test. **(D)** Brains of recipient mice were harvested on day 13 (average peak disease). Then, 40 µm thick cerebral sections were prepared and infiltrating T cells were visualized with CD3 immunohistochemistry and hematoxylin/eosin staining. Arrows depict isolated CD3^+^ T cell infiltration. Scale bar indicates 100 µm.

In order to assess the potential of IL-35^+^ DCs to abrogate autoimmune T cell activity *in vivo*, adoptive transfer EAE was induced as described in Section “Animals and Methods.” Adoptive transfer allowed us to introduce IL-35^+^ DCs into the experimental system either already during priming of the encephalitogenic lymphocytes (experimental groups 2 + 3) or only in already diseased recipient mice (experimental groups 4 + 5) (Figure 7A). After *in vivo* priming, T cells were restimulated *in vitro* as depicted in Figure 7A. T cells restimulated with myelin oligodendrocyte glycoprotein-35–55 (MOG_35–55_)-pulsed IL-35^+^ DCs (experimental group 2) exhibited a less activated phenotype and produced less IFN-γ than T cells restimulated in the presence of MOG_35–55_-pulsed mock DCs (experimental group 1) (Figure 7B). After *in vitro* restimulation, 5 × 10^6^ T cells were transferred into wild-type C57BL/6 mice. The animals were surveilled daily for the development of EAE-related symptoms. Recipient mice receiving MOG_35–55_ reactive T cells that were primed in the presence of mock DCs (experimental group 1) reproducibly exhibited EAE symptoms within 9 days after T cell transfer. A maximum disease score of three, corresponding to a complete paralysis of the hind limbs, could be observed between 14 and 18 days after T cell transfer. The animals subsequently recovered and no disease symptoms could be observed 25 days after the transfer of immunogenic T cells. By contrast, mice that received T cells primed and restimulated in the presence of MOG_35–55_ peptide loaded IL-35^+^ DCs exhibited a drastically reduced disease severity (experimental group 2). Animals injected with unpulsed IL-35^+^ DCs (experimental group 3) exhibited an intermediate disease course: EAE symptoms were less vigorous when compared to the mock DC control group but were significantly more severe than in MOG_35–55_-pulsed IL-35^+^ DC injected mice (Figure 7C). We further assayed whether the therapeutic transfer of IL-35^+^ DCs is sufficient to limit the function of MOG-primed lymphocytes. Both, *in vitro* restimulation with MOG_35–55_-pulsed IL-35^+^ DCs (experimental group 4) or the injection of IL-35^+^ DCs into recipient animals 6 and 8 days after the transfer of MOG-primed T cells (experimental group 5) slightly delayed the disease onset and led to a significant reduction of EAE symptoms.

The brains were collected at the peak of disease. Histological analysis of sections of the cerebral cortex revealed focal inflammatory lesions within the myelin rich regions with a preferential perivascular accumulation of CD3^+^ cells in diseased control animals. No formation of such lesions, but only few isolated T cells could be observed in the IL-35 experimental group (Figure 7D).

## Discussion

Due to their central role in determining the outcome and quality of adaptive immune responses, DCs are of special interest as potential therapeutic tools for the specific treatment of autoimmune disease. In contrast to the other members of the IL-12 cytokine family, IL-35 has been described to inhibit T and B lymphocyte proliferation and induce Foxp3^−^, IL-35-secreting Treg cells (iTr35) (14). Recent studies indicate that IL-35 also regulates effector functions of plasma cells as well as monocyte-derived DCs (18). Although IL-35 seems to contribute essentially to the maintenance of immune tolerance and represents a promising candidate for immunotherapy, its functionality and tolerogenic potential remain poorly investigated.

Recombinant IL-35 has a limited stability, which complicates the investigation of this cytokine (19). We thus generated a CD8α^+^ MuTu DC cell line constitutively expressing a single-chain IL-35 construct. The ectopic expression of IL-35 by the MuTu DC line efficiently interfered *in vitro* as well as *in vivo* with CD4^+^ T lymphocyte proliferation and function. A similar effect could be observed when IL-35^+^ DC were cultured with CD8^+^ T cells. Interestingly, while comparable OT-I and OT-II proliferation was observed upon the co-injection with antigen-pulsed IL-35^+^ DC *in vivo*, the number of T cells isolated from the mice was greatly diminished. This result suggests that IL-35^+^ DCs might not only affect T cell proliferation but also interferes with the survival of the target T cells. DCs have indeed been described to mediate apoptosis in activated T cells through cytokine regulated expression of Fas ligand (20). As discussed below, we assumed the observed effects on T cells might not only be a direct effect of IL-35 cytokine produced by the transgenic DC line. We furthermore hypothesized additional, IL-35 induced effects on the DC itself. IL-35^+^ DCs induced the expression of *P35* and *Ebi3*, the two subunits forming IL-35, by the interacting T cells. Similar to the findings by Collison et al. (14), the IL-35^+^ DC-conditioned T cells acquired a regulatory phenotype as shown by their ability to suppress the proliferation of CD4^+^ T cells. These results provide solid evidence for the functional expression of the IL-35 transgene by the DC line. It further supports previous findings that IL-35 does not only impair proliferation and effector functions of CD4^+^ but also CD8^+^ T cells. Interestingly, we observed that IL-35^+^ DCs but neither IL-35 containing medium nor IL-35-conditioned DCs alone induced the transcription of IL-35 subunits by CD4^+^ T cells. Moreover, in the absence of (co-)stimulation, IL-35-conditioned T cells were not capable to suppress naïve target T cell proliferation. These results suggest that IL-35 cytokine secretion alone is sufficient to regulate T cell proliferation and function. However, the induction of IL-35-expressing iTr35 cells depends on both, TLR stimulation and additional stimuli provided by DCs. Others have consistently reported that the generation of functional iTr35 is dependent on CD3/CD28-mediated activation (14, 21). Furthermore, CD80/CD86 expression by DCs is necessary for the maintenance of Treg cells (22). In the context of IL-35-mediated propagation of immune tolerance, IL-35-conditioned DCs might exert a similar function on Treg cells. We indeed observed a consistent upregulation of CD80 when DCs were conditioned with IL-35. CD80 upregulation was consistently observed, even when DCs were not stimulated by TLR ligands. We thus suggest that IL-35 contributes to a semi-mature, tolerogenic DC (tolDC) phenotype. Low level of antigen presentation, the increase in CD80 expression, together with the expression of IL-10 might be involved in the generation of iTr35 cells. We have shown in this manuscript that IL-35-conditioned DCs acquire tolerogenic functions even when IL-35 is removed but without IL-35 they cannot induce IL-35 expression in responding T cells. However, the molecular basis for this regulatory circuit has yet to be elucidated.

Immature or induced tolDCs play an essential role in the maintenance of peripheral tolerance by modulating T cell activation and inducing Tregs (23). Several mediators like IL-10 or vitamin D_3_ have been demonstrated to induce a tolerogenic phenotype on DCs. tolDCs are characterized by low levels of MHCII and costimulatory markers, as well as a reduced secretion of pro-inflammatory cytokines while expressing tolerogenic molecules such as IL-10 or IDO. In addition, the expression of CD11b has been associated with a tolDC phenotype (24, 25). In our hands, the presence of IL-35 induced a CD11c^lo^ CD11b^hi^, MHCII^lo^ semi-mature phenotype on the MuTu DC line. The presence of IL-35 impaired their ability to upregulate the expression of MHCII and costimulatory molecules in response to stimulation with TLR ligands. TLR ligand activated IL-35^+^ DCs expressed the inhibitory cytokine IL-10 whereas IL-12 secretion was greatly diminished. A similar shift in cytokine production was described by others when CD4^+^ T cells were activated in the presence of recombinant IL-35 (26). The incubation of TLR ligands activated DCs with IL-35 containing but not control culture medium had a similar effect on the expression levels of CD11c, CD11b, MHCII, and costimulatory receptors. These finding suggest that the IL-35 cytokine directly affects the MuTu DCs, maintaining them in a semi-mature state and rendering them unresponsive to TLR-activation. In contrast to IL-35^+^ DCs, no IL-10 secretion was detectable when DCs were cultured with IL-35 containing medium. This observation implies functional disparities between stably transduced and IL-35-conditioned DCs which can hardly be addressed with our current experimental setup. It is conceivable that IL-35 induced IL-10 expression requires prolonged signaling or needs to exceed a certain signal threshold. We can therefore also not completely rule out that IL-35 induces the expression of secondary, unknown mediator(s) which, in turn, drive the observed phenotypic changes of the DCs. However, a potential IL-35 receptor has been postulated to consist either of IL-12Rβ2 and IL-6 or IL-27Rα, respectively. Engagement of the receptor was demonstrated to result in the activation of STAT1-, STAT3-, or STAT4-mediated signaling in T or B cells, respectively (27, 28). It was recently shown that IL-35 interferes with the LPS-induced activation of MAPK–AP1 pathway in mouse endothelial cells, thereby suppressing the expression of various pro-inflammatory cytokines and chemokines (29). The potential receptor chains are expressed by CD8α^+^ DCs as well as in most conventional DC subtypes (our unpublished data). Thus, a direct effect of IL-35 on DCs is at least possible. In addition, both, STAT3 was well as p38/MAPK-mediated signaling, has been shown to regulate MHCII as well as costimulatory receptor expression upon DC activation. These molecular pathways therefore represent potential mediators of IL-35-induced signaling in DCs (30, 31). Further investigation is needed to elucidate the composition and expression of the IL-35 receptors on DCs as well as the downstream signaling events driving the observed phenotypic changes in IL-35-transduced and IL-35-conditioned DCs.

The IL-35 induced tolerogenic state on wild-type DCs translated functionally into an impaired capacity to stimulate antigen-specific CD4^+^ and CD8^+^ T cell responses. As proposed for other tolDC populations, the immature phenotype is characterized by low expression of MHCII and costimulatory receptors as well as the expression of suppressive molecules like IL-10. The addition of IL-10 was described to enhance the conversion of naïve CD4^+^ T cells into iTr35, representing a potential mechanism by which IL-35-conditioned DCs contribute to infectious tolerance (14). Human dexamethasone-induced tolDCs have been described to express both IL-35 subunits and these are required to elicit their full tolerogenic potential (32). It might therefore be possible that DCs can be stimulated to produce IL-35 to contribute directly immune tolerance. However, the stimuli that trigger IL-35 production, aside from IL-35 itself, remain poorly understood. In our hands, the conditioning of MuTu DCs with IL-35 did not lead to an induction of IL-35-associated genes (unpublished observations).

In order to further study the function of the IL-35 induced tolDC phenotype, we employed the IL-35^+^ DC line in two, differently immunogenic tumor models. Vaccination with tumor lysate pulsed, IL-35-expressing DCs notably accelerated tumor growth. This coincided with a reduced tumor infiltration of CD4^+^ and CD8^+^ T cells and the inhibition of their effector functions. A significant acceleration of tumor growth was only observed when the IL-35^+^ DCs were pulsed with tumor cell lysate. These observations suggest the inhibition of T cell responses to be not only caused by the systemic delivery of IL-35 but also by the interaction of antigen-presenting tolDC and tumor-specific T cells. Tumor growth in lymphocyte deficient RAG2^−/−^ animals was accelerated when compared to wild-type animals both upon mock and IL-35^+^ DC vaccination, confirming the involvement of lymphocytes in the CMT93 cancer model. In the absence of lymphocytes, tumor lysate-pulsed IL-35^+^ DC vaccination surprisingly led to an even faster tumor growth (data not shown). In accordance with Wang and colleagues, this indicates that IL-35-mediated pro-tumor effect is not solely due to suppression of adaptive immunity (6). Our observations and the EAE experiments discussed below clearly show that the effect of IL-35 is, at least partly, contact dependent. They furthermore imply that the observed effects are not merely an effect of reduced MHC class II expression.

The administration of tolerogenic cytokines is thought to re-establish antigen-specific tolerance through the prevention of autoimmune T cell reactivity and expansion of regulatory lymphocyte populations. Others have shown the treatment with recombinant IL-35 or the ectopic expression of the cytokine by different cells to protect mice against a variety of autoimmune diseases (1, 33, 34). Furthermore, the intraperitoneal injection of IL-35 was recently shown to ameliorate autoimmune pathogenicity in a murine asthma model partly through the induction of an immune-suppressive phenotype on monocyte-derived DCs (18). We therefore investigated whether the IL-35^+^ MuTu DC line might be employed to affect the development of the CNS autoimmune disease EAE. The preventive transfer IL-35^+^ DCs but not control DCs was found to significantly reduce EAE symptoms. The observed effect was found to be mediated through the impairment of activation and function of the transferred CD4^+^ T cells, resulting in a reduction of CNS infiltration and inflammation. This can be only partially attributed to the systemic delivery of IL-35 by the IL-35^+^ DCs itself, as the transfer of unpulsed IL-35^+^ DCs indeed reduced EAE severity. However, a further, significant reduction of EAE severity was observed only upon loading the IL-35^+^ DCs with myelin specific peptide. These findings suggest the interaction of IL-35^+^ DCs and T lymphocytes to be required for an optimal suppression of autoimmunity. Thus, the IL-35 induced immature phenotype of the DCs contributes to the observed reduction of EAE severity. We therefore propose IL-35-conditioned CD8α^+^ DCs to contribute indirectly to the IL-35-mediated regulation of adaptive immune responses *via* the adaption of an immature phenotype. We further sought to determine the latest time point at which the application of MOG-pulsed IL-35^+^ DCs is capable of affecting EAE development. *In vitro* restimulation of MOG-primed T lymphocytes with IL-35^+^ DC and also the injection of IL-35^+^ DCs into recipient mice significantly reduced EAE severity. Thus, DC transfer, together with the delivery or ectopic expression of IL-35 may represent a potential preventive and also therapeutic tool for the regulation of pathologic (auto-) inflammation. Considering the particular tolerance mechanisms employed by IL-35, the combination with other tolerogenic molecules, such as TGF-β or IL-10, might potentiate immunosuppressive effects in therapeutic settings.

Taken together, our data indicate that IL-35^+^ MuTu DCs are able to exert regulatory functions on CD4^+^ and CD8^+^ T cells. More importantly, IL-35 was shown to induce a tolerogenic phenotype on CD8α^+^ MuTu DCs, thereby indirectly contributing to IL-35-mediated immune regulation. Our work adds a novel aspect to the functions of this poorly investigated cytokine. Further investigation of IL-35 induced effects on DC function, and also on other innate immune cells’ function may help us to better understand IL-35-mediated immune regulation and to exploit its potential to regulate immune responses.

## Ethics Statement

The study was approved by the local, cantonal, and Swiss veterinary offices. Permit no. VD2490.1.

## Author Contributions

SH performed analysis on all samples, interpreted data, and wrote manuscript. AD, RM, and MS performed experiments and helped with data analysis. HA-O supervised the work, helped in data interpretation, manuscript evaluation, and acted as corresponding author.

## Conflict of Interest Statement

The authors declare that the research was conducted in the absence of any commercial or financial relationships that could be construed as a potential conflict of interest.

## Abbreviations

actTGF-β, active TGF-β; DC(s), dendritic cell(s); MuTu DC, murine tumor DC line; iTr35, IL-35-expressing regulatory T cell.
