# Supplementary material for: Interleukin-35-Producing CD8α+ Dendritic Cells Acquire a Tolerogenic State and Regulate T Cell Function
Source: Front Immunol. 2017 Feb 8;8:98. doi: 10.3389/fimmu.2017.00098 (PMC5296329; doi:10.3389/fimmu.2017.00098)
Supplement: Supplementary file 1 [file image_1.pdf]

Supplementary Material

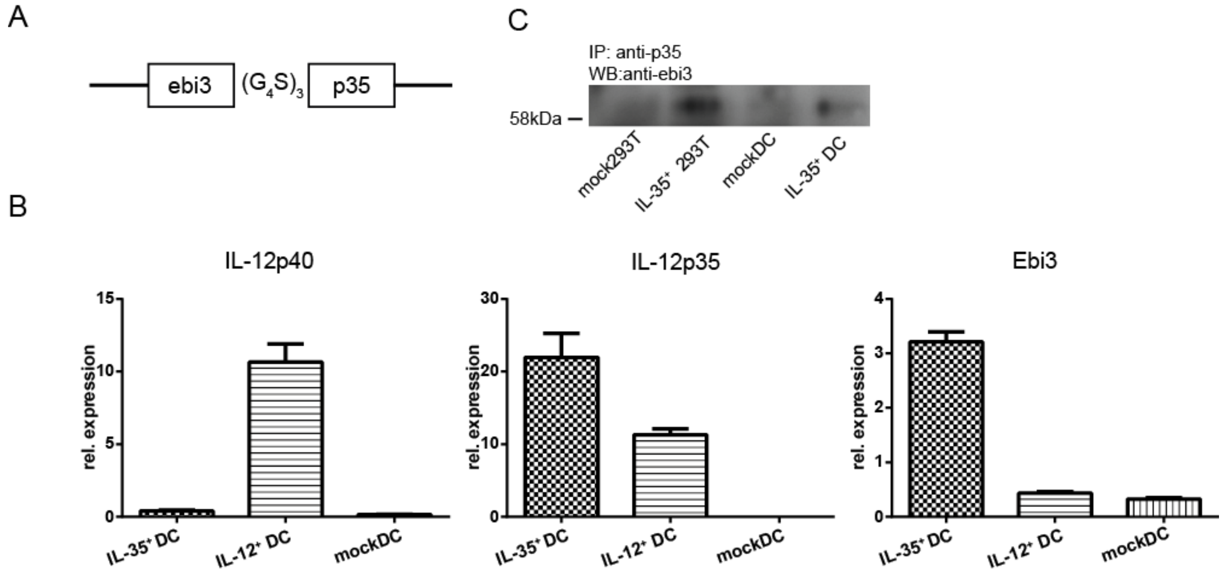

Supplementary Figure 1 Generation and verification of a constitutively IL-35 expressing MuTu dendritic cell line. A) Schematic overview on the lentiviral expression construct comprising the IL-35 chains Ebi3 and Il-12p35 connected by a flexible (Gly$_4$Ser)$_3$ linker allowing constitutive expression of the cytokine in form of a single-chain fusion protein. Transcription of transgene construct was verified by B) real-time PCR using specific primers for the IL-35 subunits Il-12p35 and Ebi3. C) Transduced MuTu DC and 293T HEK cells were precipitated using an Il-12p35 specific antibody and detected by western blot using an Ebi3 specific antibody. The cDNA of the fusion protein was also detectable by real-time PCR using llinker and p35-spanning primers.

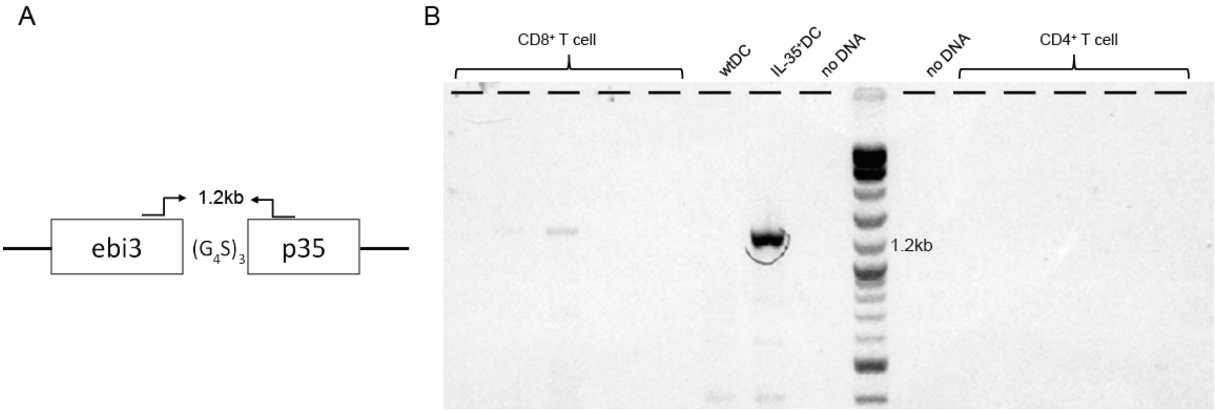

<u>Supplementary Figure 2</u> IL-12p35 and Ebi3 transcription detected in the MLRs did not stem from single-chain IL-35 as expressed by the transgenic IL-35$^+$ DC line: A) In order to exclude the possibility of a contamination of the analyzed T cell RNA by IL-35$^+$ DC, T cells were sorted. The respective cDNA was isolated and subjected to conventional PCR using primers amplifying only IL-35$^+$ DC derived single-chain IL-35 but not the induced endogenous cytokine consisting of two independent protein chains. B) While amplification of IL-35$^+$ DC derived cDNA yielded the PCR product of the expected size, none or only faint single-chain IL-35 could be detected in T cell/DC co-culture cDNA.

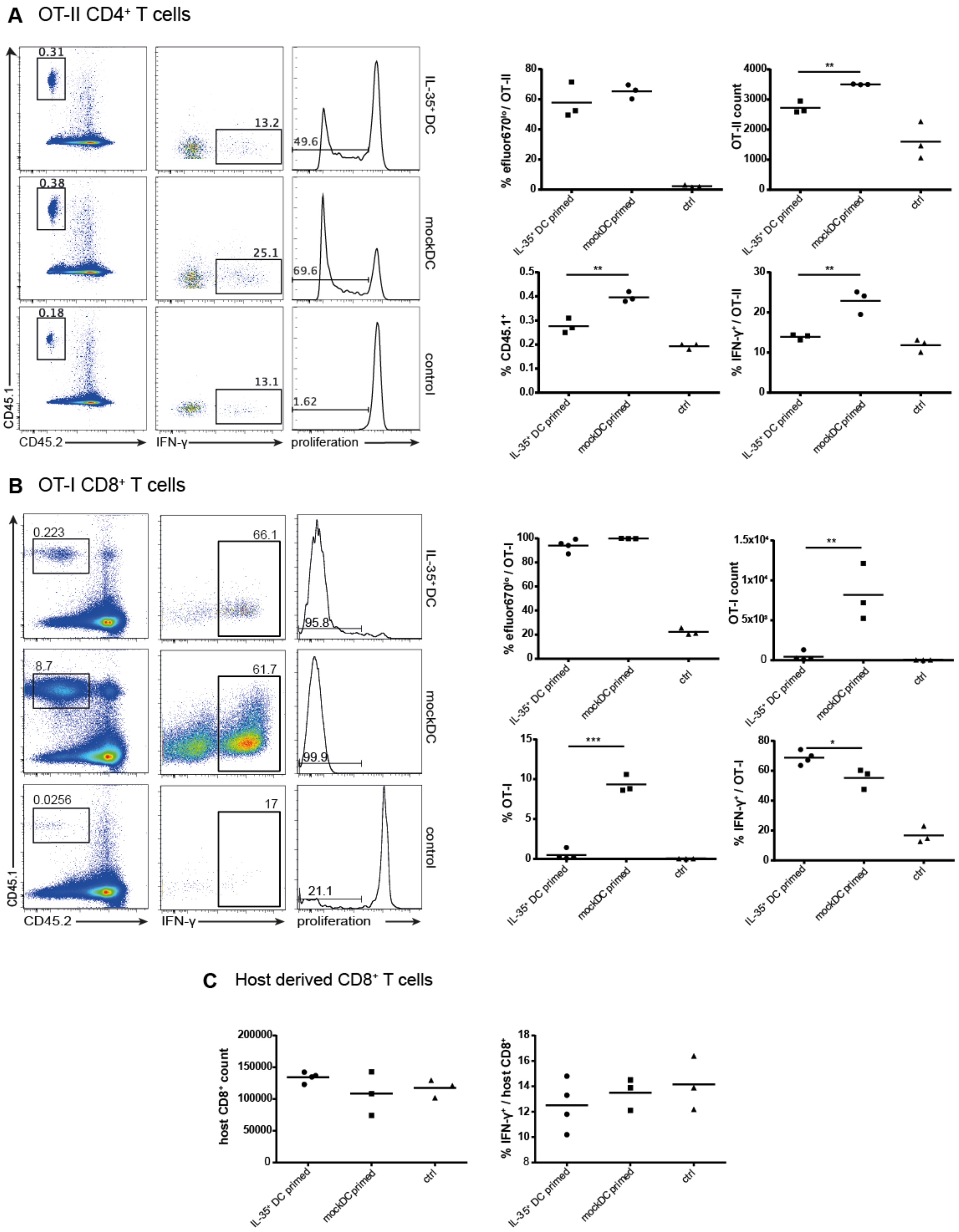

<u>Supplementary Figure 3</u> Il-35 expressing dendritic cells impair antigen-specific T cell proliferation and function *in vivo*. Wild-type C57BL/6 mice (n=3 per experimental group) were intravenously injected with $2x10^5$ $CD45.1^+$ $CD4^+$ OT-II T cells (A) or $CD45.1^+$ $CD8^+$ OT-I T (B and C) cells and $2.5x10^6$ $OVA_{323-339}$ (OT-II, 50nM) respectively SIINFEKL (OT-I,

1μM) peptide pulsed mock transduced or IL-35$^+$ MuTu DC. After four days, spleen cells were isolated and T lymphocytes analyzed by flow cytometry. A) Analysis of CD45.1$^+$ OT-II cell exhibited only a moderate decrease of proliferation and total cell number but a significant reduction of IFN-γ production when co-injected with IL-35$^+$ DC but not upon co-transfer of mockDC. B) Co-injection of IL-35$^+$ DC led to a drastic reduction of OT-I CD8$^+$ T cells that could be isolated form the respective animals. However, the few remaining OT-I T cells exhibited proliferation and IFN-γ production comparable to cells isolated from mock transduced control DC co-injected animals. C) Host derived CD45.2$^+$ CD8$^+$ T cell count and IFN-γ expression was found to be similar in all three experimental groups. Graphs show representative data from one of three independent experiments each.

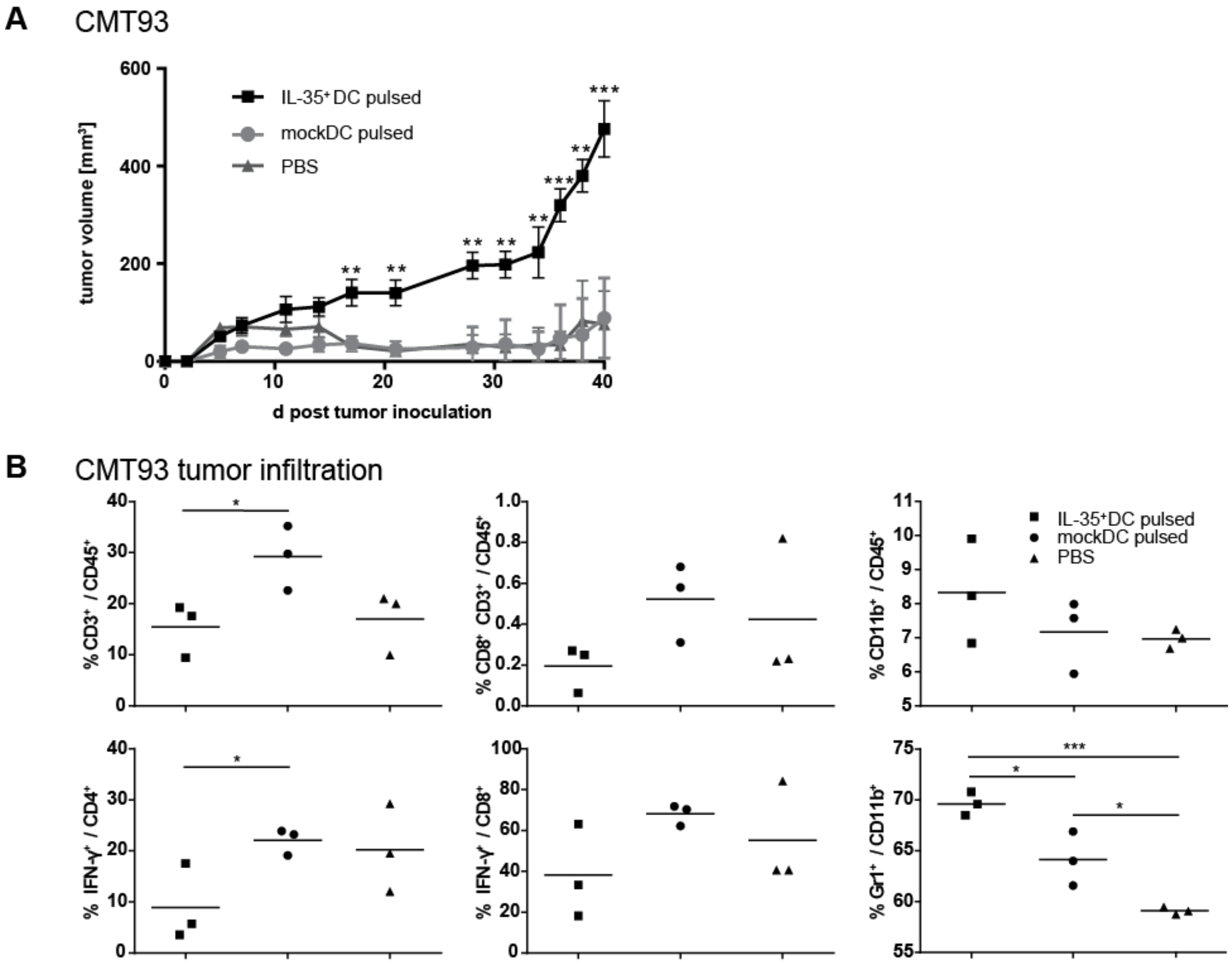

Supplementary Figure 4 IL-35 expressing DC promote tumor growth. A) C57BL/6 mice were intravenously vaccinated a total of three times with 2.5x10^6 tumor cell lysate pulsed, IFN-γ activated IL-35$^+$ DC, mock transduced DC or PBS. 2x10^6 CMT93 carcinoma cells were transferred subcutaneously in the flank of the animals. B+C) Tumor growth was followed and measured regularly using a caliper. Results are expressed as the mean of tumor volumes ± SD from three experiments (n=3 per experimental group) each. D) Tumors were resected 12 days after inoculation and enzymatically digested. The isolated cells restimulated and tumor infiltrating cells were analyzed by flow cytometry. Data denoted as mean ± SD. The data shows representative results from one of three independent experiments (n=3 per experimental group)
